# Allopregnanolone relieves paclitaxel induced mechanical hypersensitivity via inhibiting spinal cord PGE_2_-EP2 mediated microglia-neuron signaling

**DOI:** 10.1016/j.ibneur.2025.01.011

**Published:** 2025-01-16

**Authors:** Kunlin Guo, Lei Gao, Ping Li, Shanwu Feng, Liping Zhao, Xian Wang

**Affiliations:** Department of Anesthesiology, Women’s Hospital of Nanjing Medical University, Nanjing Women and Children’s Healthcare Hospital, Nanjing, Jiangsu, China

**Keywords:** Chemotherapy-induced neuropathic pain, Paclitaxel, Allopregnanolone, Spinal cord, PGE_2_, Microglial

## Abstract

Chemotherapy-induced neuropathic pain (CINP) is a serious adverse effect of commonly used chemotherapeutics. Neurosteroid allopregnanolone is suggested to modulate the expression of various receptors or enzymes that involved in pain perception, presenting an analgesic potential. Here, we investigated if allopregnanolone attenuates extracellular signal-regulated kinase (ERK) and its downstream prostaglandin E_2_ (PGE_2_) expression in the dorsal spinal cord concomitant with neuropathic pain relief in paclitaxel (PTX)-induced neuropathic pain model rats. The results showed PTX upregulated phosphorylated ERK (p-ERK), PGE_2_ level, and PGE_2_ receptor E-prostanoid 2 (EP2) expression in the spinal dorsal horn. Besides, p-ERK inhibitor PD98059 or microglia inhibitor minocycline reduced microglial activation, p-ERK expression, PGE_2_ release, EP2 expression, and partially alleviated PTX-induced mechanical hypersensitivity. Further, allopregnanolone level in the dorsal spinal cord was observed to decrease in CINP rats, and intragastric administration of exogenous allopregnanolone dose-dependently alleviated PTX-induced mechanical hypersensitivity. Mechanistically, allopregnanolone dose-dependently alleviated PTX-induced microglial activation, p-ERK, PGE_2_, and EP2 upregulation, as well as cytokines expression in the dorsal spinal cord in CINP rats. Furthermore, subcutaneous injection of allopregnanolone synthesis inhibitor medroxyprogesterone could reduce endogenous allopregnanolone and block all effects of exogenous allopregnanolone in CINP rats. Taken together, these results suggest allopregnanolone presents an analgesic effect for PTX-induced mechanical hypersensitivity, partially via inhibiting the dorsal spinal cord PGE_2_-EP2 mediated microglia-neuron signaling.

## 1 Introduction

Chemotherapy-induced neuropathic pain (CINP) is a severe side effect that occurs in up to 80 % of cancer survivors during treatment with chemotherapeutic drugs such as paclitaxel (PTX), oxaliplatin, and vincristine (Sisignano et al., 2014). It is characterized by numbness, burning, tingling pain, heat and cold pain and has a ‘stocking and glove’ distribution (Staff et al., 2020). This side effect can significantly decrease the life quality of survivors, limit the tolerable dose of chemotherapeutics, and even induce chemotherapy discontinuation (Farquhar-Smith, 2011). PTX is one of the most commonly used chemotherapeutic drugs and is usually used to treat ovarian, breast, cervical, and lung cancers (Vyas and Kadow, 1995). Although highly effective in blocking tumor development, PTX produces CINP in as high as 60–70 % patients (Staff et al., 2020). Moreover, drugs used for chronic pain, such as opioids, anticonvulsants, or antidepressants, present little or no analgesic effect for CINP in preclinical or clinical trials (Sisignano et al., 2014). Therefore, it is necessary to explore the mechanism and alternative treatment for CINP.

Allopregnanolone is a cholesterol-derived neurosteroid to produce sedative, amnesic, anti-anxiety, and anti-depressive effect (Paul et al., 2020, Porcu et al., 2016). In recent years, certain preclinical studies have suggested the analgesic potential of allopregnanolone for various types of pain conditions. For example, in rats with chronic temporomandibular joint inflammation, allopregnanolone can quickly alleviate mechanical allodynia (Hornung et al., 2020). Additionally, allopregnanolone can alleviate hyperalgesic behavior following diabetes or nerve injury (Afrazi et al., 2014). Moreover, allopregnanolone can prevent and suppress the painful neuropathy induced by oxaliplatin (Meyer et al., 2011). Although allopregnanolone modulates various membrane receptors or ion channels that distributes in the pain regulatory pathway, such as GABA_A_ and glycine, as well as L-and T-type calcium channels, in either autocrine or paracrine manner (Patte-Mensah et al., 2014). However, mechanisms underlying CINP relief by allopregnanolone remain unclear.

Previous studies have shown that the activation of mitogen-activated protein kinases (MAPKs) in the dorsal root ganglion (DRG) and spinal dorsal horn contributes to the nociceptive response after nerve injury and/or inflammation (Obata and Noguchi, 2004). MAPK is a family of serine/threonine protein kinases that includes extracellular signal-regulated protein kinase (ERK), c-Jun N-terminal kinase, and p38-MAPK (Johnson and Lapadat, 2002). ERK activation occurs in rats’s ipsilateral dorsal horn, DRG, and gracile nucleus following chronic constriction injury, spinal nerve ligation, peripheral nerve injury, or partial sciatic nerve ligation (Ma and Quirion, 2005). Dr. Maruta and his team found that upregulated p-ERK in DRG was associated with oxaliplatin-induced neuropathic pain, and that intrathecal administration of the ERK inhibitor PD98059 could improve mechanical pain (Maruta et al., 2019). In addition, there is evidence that p-ERK is an upstream regulator of PGE_2_ release. PGE_2_ is associated with the induction of central sensitization in spinal cord neurons (Zhao et al., 2007). PGE_2_ and its receptor EP2 play an essential role in central inflammatory hyperalgesia (Akundi et al., 2005, Reinold et al., 2005, Hösl et al., 2006).

Collectively, we raised the question of whether spinal cord p-ERK and its downstream PGE_2_-EP2 signaling participated in the analgesic effect of allopregnanolone on CINP. Accordingly, we firstly established the CINP rat model and examined the expression of p-ERK, PGE_2_, EP2 in the dorsal spinal cord in CINP rats. Endogenous allopregnanole expression was detected as well. Further, after treatment with the p-ERK inhibitor PD98059 and the microglia inhibitor minocycline, we examined p-ERK, PGE_2_, EP2 expression, as well as pain behavior in CINP rats. Furthermore, p-ERK, PGE_2_, EP2 expression, cytokines content, as well as pain behavior were examined after treatment with different doses of exogenous allopregnanolone or the allopregnanolone inhibitor medroxyprogesterone in CINP rats.

## 2 Materials and methods

### 2.1 Animals preparation

With approval of the Institutional Animal Care and Use Committee of Nanjing Medical University (approval no. IACUC-2310016), adult male Sprague-Dawley rats (180–220 g) were used. Rats were housed individually with adequate food and water and acclimatized to the environment (temperature, 23–25 ˚C; humidity, 40–60 %; a 12-/12-h light/dark cycle) for 7 days before the experiment. Finally, a total of 96 rats were used in our study. Of note, females were not used due to the fluctuation of endogenous progesterone that may interfere with the results.

### 2.2 Study design and drug treatment

We firstly established CINP rat model with our previous method (Wang et al., 2022). In brief, PTX (TargetMol, T0968) was dissolved in dimethyl sulfoxide (DMSO, 0.5 mg/ml) and intraperitoneally (i.p.) injected at a dose of 2 mg/kg at alternate days 0, 2, 4, 6, resulting in a cumulative dose of 8 mg/kg. The animals were allowed to survive for 1, 3, 7, and 14 days, respectively. Control rats received an equal volume of DMSO. At predertermined time points, we detected the mechanical withdrawal threshold (n = 6 for each time point). Afterwards, the rats were sacrificed and processed for western blot, immnuofluoresence, double immunofluorescence staining, and enzyme-linked immunosorbent assay (ELISA).

To explore the etiological role of glial p-ERK expression in mechanical hypersensitivity after PTX treatment, rats were allocated to the following groups: Con, CINP, CINP + vehicle, CINP + PD98059 and CINP + minocycline group (n = 6 for each group). PD98059 (2′-amino-3′-methoxyflavone, TargetMol, T2623) or minocycline (Targetmol, T1101) was intrathecal (i.t.) injected for targeted inactivation of ERK1/2 and inhibition of spinal cord microglia activation, respectively. In brief, for rats in CINP + PD98059 group, PD98059 was dissolved in DMSO (10 mg/ml) and i.t. injected at a dose of 10 μg daily for three consecutive days, with the first injection beginning on day 8 after PTX injection based on previous report (n = 6) (Lim et al., 2003, Zhuang et al., 2005). For rats in CINP + minocycline group, minocycline was dissolved in 0.9 % normal saline and i.t. injected at a dose of 100 μg daily for three consecutive days, with the first injection beginning on day 8 after PTX injection (n = 6). For rats in CINP + vehicle group, an equal volume of DMSO was used as vehicle (n = 6). At 7, 8, 9, 10, 12 and 14 day after PTX treatment, we detected the mechanical withdrawal threshold. And after the final pain behavioral testing, the rats were euthanatized and processed for western blot and ELISA.

Spinal cord allopregnanolone expression was examined with ELISA on days 0, 3, 7, and 14 after PTX treatment. To observe the potential analgesic effect of allopregnanolone, exogenous allopregnanolone (cat. no. A878696; MACKLIN) was dissolved in ethanol, further diluted in lactated ringer’s solution to a final concentration of less than 1 %, and administered to rats via orogastric tube at doses of 5, 10, or 20 mg/kg once a day (n = 6 for each dose) on days 1, 3, 5, 7, 9, 11, and 13 after PTX treatment (Huang et al., 2016). An equal volume of ethanol was used as vehicle solution (n = 6). We detected the mechanical withdrawal threshold on days 0, 3, 7, and 14 after PTX treatment. And after the final pain behavioral testing, the rats were euthanatized and processed for western blot and ELISA.

As medroxyprogesterone could inhibit the conversion of dihydroprogesterone to allopregnanolone, we administered exogenous medroxyprogesterone in rats to further decrease endogenous allopregnanolone level and to observe the analgesic effect of allopregnanolone. In control and CINP rats, medroxyprogesterone (cat. no. M830019; MACKLIN) was suspended in olive oil and injected subcutaneously in the subscapular area at doses of 10 or 20 mg/kg in a volume of 1 ml/kg on days 1, 3, 5, 7, 9, 11, and 13 after PTX treatment (n = 6 for each group) (Huang et al., 2016). Behavioral testing was performed on these rats at day 14. Afterwards, the rats were euthanatized and processed for western blot and ELISA.

### 2.3 Nociceptive behavioral testing

The mechanical withdrawal threshold was detected using the electronic pain detector (Yuyan, Shanghai, BME-404). Briefly, rats were placed individually to adapt to the environment for 30 minutes. Then, the electronic pain detector was used to measure the paw withdrawal threshold of rats via slightly touching the hindpaw with a gradual increased intensity from 0 to 80 g. The mechanical withdrawal threshold was recorded and averaged from the 6 animals in each group.

### 2.4 Western blot analysis

For western blot analysis, the rats were deeply anesthesized using 6–8 % sevoflurane, and the L4–5 spinal cord segments was collected. The dorsal spinal cord tissues were homogenized in ice-cold RIPA lysis buffer (cat. no. bl504a; Biosharp Life Sciences), and the supernatant was collected after centrifugation at 10000 g for 20 min at 4 ˚C. Protein concentration was determined using a BCA protein assay kit (cat. no. bl521a; Biosharp Life Sciences). Subsequently, 30 μg of protein from each sample was resolved on a 12 % SDS-PAGE gel and transferred to polyvinylidene difluoride (PVDF) membrane. The membrane was blocked with 5 % bovine serum albumin (BSA) for 1 h at room temperature (RT) and then incubated over night at 4 ˚C. The primary antibodies used were specific for p-ERK (1:1000; cat. no. ab201015; Abcam), EP2 (1:500; cat. no. DF5140; Affinity), and Iba-1 (1:500; cat. no. ab178846; Abcam). As internal controls, antibodies against ERK (1:10000; cat. no. ab184699; Abcam) and β-actin (1:1000; cat. no. GB11001; Servicebio) were employed. After three washes with tris‑buffered saline containing 0.05 % Tween-20, the PVDF membrane was incubated with horseradish peroxidase (HRP)‑conjugated secondary antibodies (1:5000; cat. no. BL003A; Biosharp) for 1 h at RT. The immunoreactive band was then developed using an ECL detection solution (cat. no. bl520a; Biosharp Life Sciences) for 30 s and visualized using a luminescent imaging system (BIORAD, ChemiDoc XRS+). The optimal density of specific protein band was analyzed using ImageJ software (v. 1.53a; National Institures of Health).

### 2.5 Immunofluorescence and double immunofluorescence staining

For immunofluoresence staining, the rats were deeply anesthetized with 6–8 % sevoflurane and transcardially perfused with 4 % paraformaldehyde. The lumbar spinal cord was carefully removed, fixed in the same fixative overnight at 4℃, and embedded in paraffin. For immunofluoresence staining, the spinal cord tissue (30 μm) was mounted on glass slides. After sequencing dewaxing, hydration, and microwave antigen repair in sodium citrate for 20 min, the sections were incubated with 3 % H_2_O_2_ and blocked with 3 % BSA for 30 min at RT. After overnight incubation with a rabbit monoclonal antibody aganist p-ERK (1:100; cat. no. ab201015; Abcam) overnight at 4 ℃, the sections were incubated with MaxVision ™ anti-rabbit HRP-Polymer (Fuzhou Maixin, KIT-5005) for 30 min at RT, and labeled using a separate cy3 tryamide (AAT Bioquest, 11065) signal amplification system. Nuclear DNA was labeled in blue with 4′,6′-diamidino-2-phenylindole (DAPI). Finally, the obtained immunofluorescence sections were scanned and stored using an OlyVIA software (Olympus VS200; Olympus Corporation).

For double immunofluorescence staining, the spinal cord sections were prepared as aforementioned and incubated in two rounds of staining. In the first round, the sections were incubated with rabbit monoclonal antibody aganist p-ERK (1:100; cat. No. ab201015; Abcam) or rabbit polyclonal antibody agansit EP2 (1:300; cat. No. DF4878; Affinity) overnight at 4 ℃. After rewarming, these sections were incubated with MaxVision ™ anti-rabbit HRP-Polymer (Fuzhou Maixin, KIT-5005) at RT for 30 min following with a cy3 tryamide (AAT Bioquest, 11065) signal amplification system. In the second round of staining, the sections were incubated for 1 h with one of the following antibodies: microglia marker rabbit polyclonal antibody against Iba-1 antibody (1:1000; cat. no. 10904–1-AP; Proteintech), neurons marker rabbit monoclonal antibody against NeuN (1:3000; cat. no. ab177487; Abcam), or astrocytes marker mouse monoclonal antibody against GFAP (1:10000; cat. no. 60190–1-lg; Proteintech). Alexa Fluor® 488 AffiniPure donkey anti-rabbit IgG (H+L) (1:400; cat. no. 711545151; Jackson ImmunoResearch) or Alexa Fluor® 488 AffiniPure donkey anti-mouse IgG (H+L) (1:400; cat. no. 711545150; Jackson ImmunoResearch) was applied at RT for 30 min based on the secondary antibody. Sodium citrate antigen retrieval was used in between two rounds of tyramide signal amplification to remove the antibody from the previous round, to avoid any cross-reactivity. Nuclear DNA was lablled in blue with DAPI. Finally, the obtained immunofluorescence sections were scanned and stored using an OlyVIA software (Olympus VS200; Olympus Corporation).

### 2.6 ELISA

The levels of allopregnanolone and pro-inflammatory cytokines in the spinal cord were determined by ELISA. The samples were prepared as that in the western blot analysis. The supernatant was collected, and the levels of prostaglandin E2 (PGE_2_), allopregnanolone, tumor necrosis factor-α (TNF-α), as well as monocyte chemoattractant protein-1 (MCP-1) were quanfified using commercially available ELISA kits (Aifang biological, AF2944-A, AF40125-A, AF3056-A, AF2975-A, respectively) according to the manufacturer’s instructions. The OD value was acquired at 450 nm using a multi-function microplate reader (Rayto RT-6100).

### 2.7 Statistical analysis

Statistical analysis was performed using GraphPad Prism software (version 8.0; GraphPad Prism, USA). Data are presented as the mean ± SEM. The mechanical withdrawal threshold in each time point between rats in control and CINP group, as well as PGE2 and EP2 expression between the two groups were compared using non-paired t test. In addition, one-way ANOVA was used to assess differences in the other western blot, immunofluorescence staining, or ELISA results among the groups. If a significant difference was observed, the Bonferroni post hoc test was applied. P＜0.05 was considered to indicate a statistical significant difference.

## 3 Results

### 3.1 Paclitaxel (PTX) treatment induces mechanical hypersensitivity in rats

**Fig. 1:**
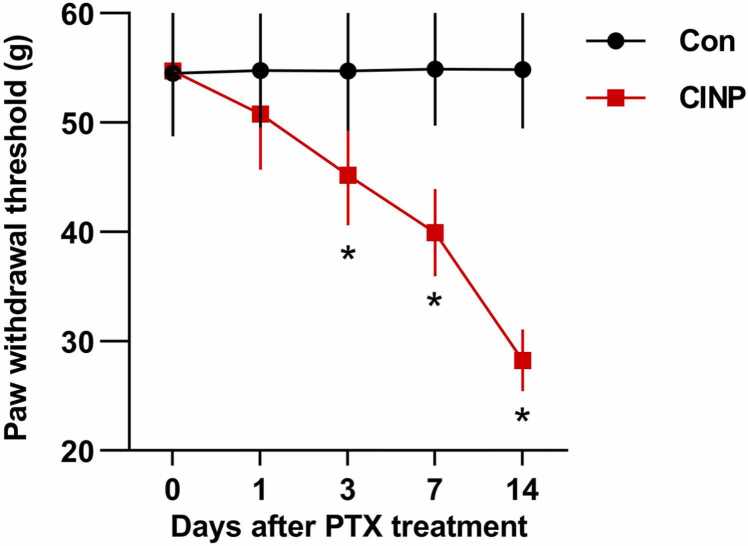
Paclitaxel (PTX) treatment induces mechanical hypersensitivity in rats. We established CINP rat model with 4 alternate i.p. injections of PTX 2 mg/kg. Control rats received an equal volume of DMSO. In each time point after PTX treatment, the mechanical withdrawl threshold was compared with non-paired t test for rats between the 2 groups. Data were represented mean ± SEM. *P＜0.05 for comparisons between rats in the 2 groups. n = 6 rats for each group.

We established CINP rat model with alternate i.p. injection of PTX 2 mg/kg for four times. As shown, a cumulative dose of PTX 8 mg/kg prominently reduced the mechanical withdrawal threshold, which appeared at day 1 after PTX treatment and peaked at day 14 (from baseline 54.75 ± 11.61 g to day 14, 28.25 ± 7.812 g). However, no obvious pain behavioral change was observed in control rats (Fig. 1).

### 3.2 PTX treatment upregulates p-ERK expression in the microglia in the spinal dorsal horn

**Fig. 2:**
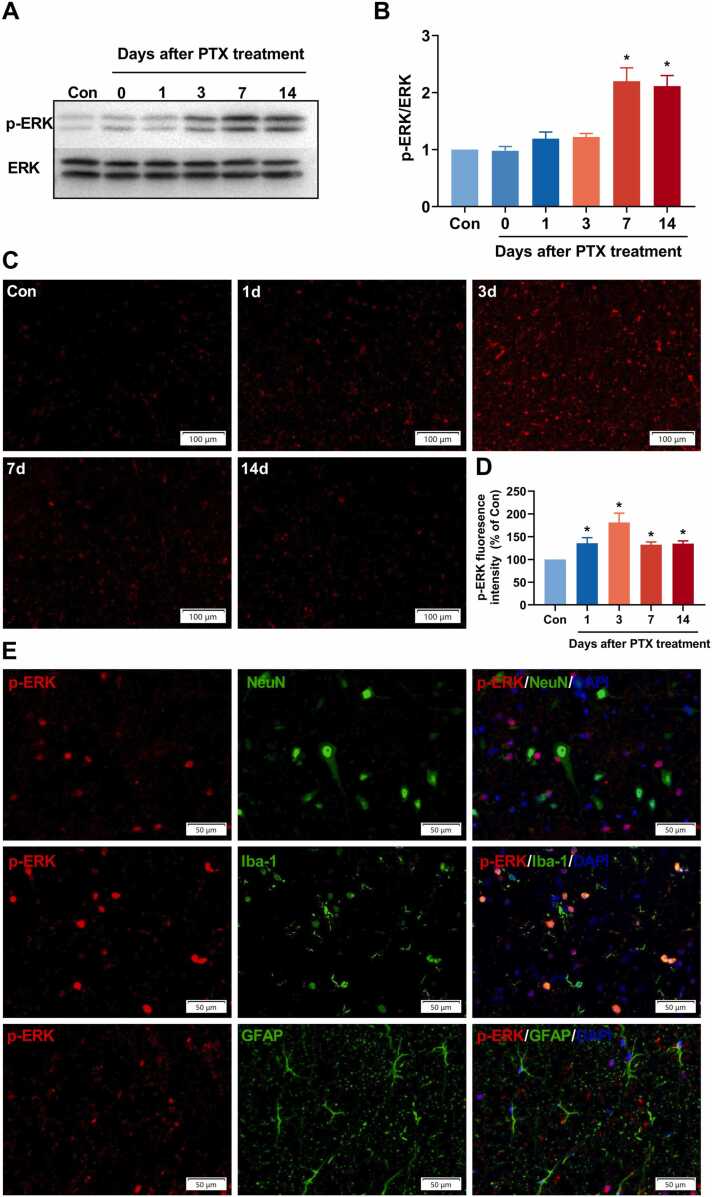
PTX treatment upregulates phosphorylated extracellular signal-regulated kinase (p-ERK) expression in the microglia in the spinal dorsal horn. We established CINP rat model with 4 alternate i.p. injections of PTX 2 mg/kg. Control rats received an equal volume of DMSO. Western blot analysis examined the expression of p-ERK in the dorsal spinal cord after PTX treatment. In addition, immunofluorescence and double immunofluorescence staining detected the expression and cellular distribution of p-ERK in the spinal dorsal horn. (A-B) Quantification of p-ERK expression in the dorsal spinal cord in control and CINP rats using western blot analysis. Compared with control rats, the p-ERK level in the dorsal spinal cord was gradually increased throughout the 14 days’ observational period and reached a peak at day 7 after PTX treatment. The ERK was used as a loading control. Data were represented mean ± SEM. *P＜0.05 compared with control rats. n = 6 rats for each group. (C-D) Immunofluorescence of p-ERK in the spinal dorsal horn in control and CINP rats. p-ERK immunoreactivity increased as early as day 1 and peaked on day 3 after PTX treatment. Data were represented mean ± SEM. *P＜0.05 compared with control rats. n = 6 rats for each group. Scale bar = 100 μm. Additionally, p-ERK expression in the spinal dorsal horn was double-stained with cell-specific markers: NeuN, Iba-1, and GFAP, respectively, at day 14 after PTX treatment. As shown, p-ERK was colocalized with Iba-1 but not GFAP or NeuN **(E)**, indicating that the phosphorylation of ERK in the spinal dorsal horn was restricted to microglial cells. Scale bar = 50 μm.

The expression of p-ERK in the dorsal spinal cord was examined with western blot and immunofluorescence staining at days 0, 1, 3, 7, and 14 after PTX treatment. As shown by western blot, p-ERK presented a low expression in control rats. However, p-ERK but not ERK expression gradually increased throughout the 14 days’ observational period and reached a peak at day 7 after PTX treatment **(**Fig. 2**A)**. Consistently, very low p-ERK immunoreactivity was detected in the spinal dorsal horn in control rats, which increased as early as day 1 and peaked on day 3 after PTX treatment **(**Fig. 2**B)**.

In addition, to identify the cellular distribution of increased p-ERK immunoractivity after PTX treatment, p-ERK expression in the spinal dorsal horn was double-stained with cell-specific markers: NeuN for neurons, Iba-1 for microglia, and GFAP for astrocytes, respectively, at day 14 after PTX treatment. We found that p-ERK was colocalized with Iba-1 but not GFAP or NeuN **(**Fig. 2**C)**, indicating that the phosphorylation of ERK in the spinal dorsal horn was restricted to microglial cells. These results suggested that p-ERK activation in the spinal dorsal horn microglial cells might participate in PTX-induced mechanical hypersensitivity in rats.

### 3.3 PTX treatment upregulates prostaglandin E_2_ (PGE_2_) level and increases the expression of the PGE_2_ receptor E-prostanoid 2 (EP2) in the spinal dorsal horn

**Fig. 3:**
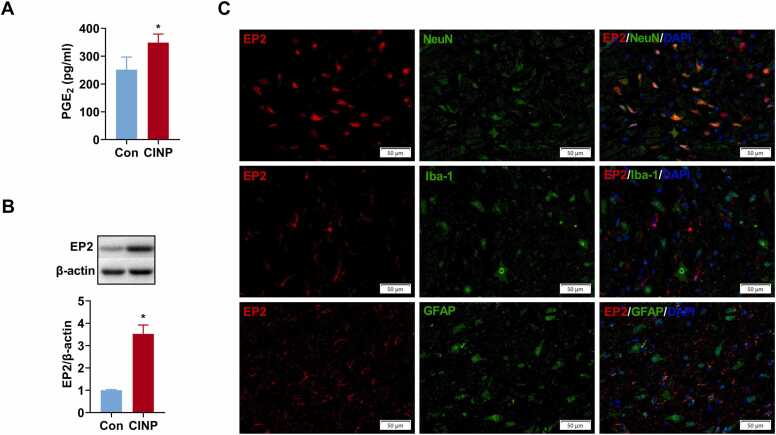
PTX treatment upregulates prostaglandin E_2_ (PGE_2_) level and increases the expression of the PGE_2_ receptor E-prostanoid 2 (EP2) in the spinal dorsal horn. We established CINP rat model with 4 alternate i.p. injections of PTX 2 mg/kg. Control rats received an equal volume of DMSO. ELISA examined the level of PGE_2_ in the dorsal spinal cord after PTX treatment. In addition, western blot and double immunofluorescence staining detected the expression and cellular distribution of EP2 in the spinal dorsal horn. (A) Quantification of PGE_2_ expression in the dorsal spinal cord in control and CINP rats using ELISA. Comparing with control rats, the release of PGE_2_ in CINP rats was significantly increased. Data were represented mean ± SEM. *P＜0.05 compared with control rats. n = 6 rats for each group. (B) Quantification of EP2 expression in the dorsal spinal cord in control and CINP rats using western blot. It showed a significant increase in EP2 expression in CINP rats compared to control rats. Data were represented mean ± SEM. *P＜0.05 compared with control rats. n = 6 rats for each group. (C) EP2 expression in the spinal dorsal horn was double-stained with cell-specific markers: NeuN, Iba-1, and GFAP, respectively, at day 14 after PTX treatment. As shown, EP2 was colocalized with NeuN but not Iba-1 or GFAP, indicating that the EP2 expression upregulation was happened in neurons. Scale bar = 50μm.

p-ERK is an upstream regulator of PGE_2_ release (Zhao et al., 2007), the level of PGE_2_ in the dorsal spinal cord was determined at day 14 after PTX treatment using ELISA. Comparing with control rats, the release of PGE_2_ in CINP rats was significantly increased (348.9 ± 30.44 pg/ml in CINP rats *vs.* 251.6 ± 45.37 pg/ml in control rats, Fig. 3**A**)**.** PGE_2_-EP2 signaling pathway is essential in central and spinal inflammatory hyperalgesia (Reinold et al., 2005, Hösl et al., 2006). Thus, we tentatively examined EP2 expression in the dorsal spinal cord. Western blot showed a significant increase in EP2 expression in CINP rats compared to control rats (Fig. 3**B)**. In addition, we examined the localization of EP2 in the dorsal spinal cord after PTX treatment. As shown in Fig. 3**C**, double immunofluorescence staining showed that EP2 was colocalized with NeuN but not Iba-1 or GFAP, indicating PGE_2_-EP2 mediated migroglia-neurons signaling might participate in PTX induced mechanical hypersensitivity.

### 3.4 p-ERK inhibitor PD98059 or microglia inhibitor minocycline reduces microglial activation, p-ERK expression, PGE_2_ release, EP2 expression, and partially alleviates PTX-induced mechanical hypersensitivity

**Fig. 4:**
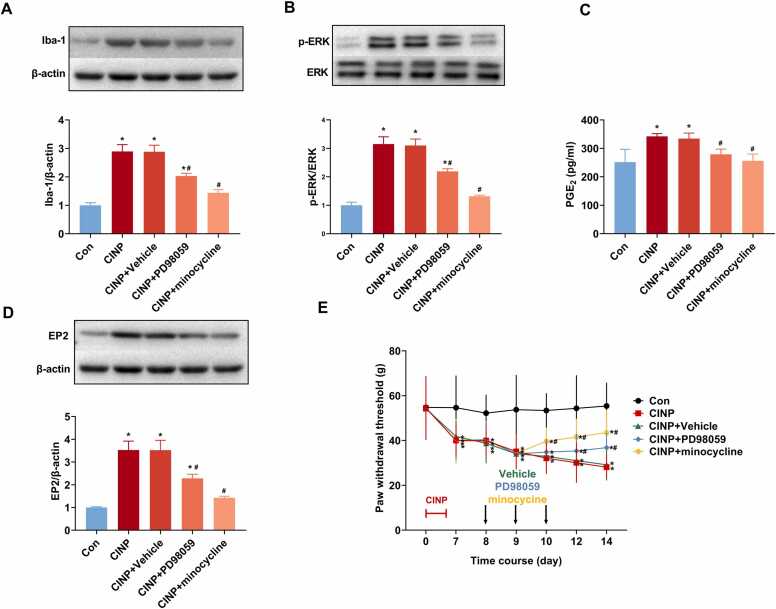
p-ERK inhibitor PD98059 or microglia inhibitor minocycline reduces microglial activation, p-ERK expression, PGE_2_ release, EP2 expression, and partially alleviates PTX-induced mechanical hypersensitivity. p-ERK antagonist PD98059 or microglia activation inhibitor minocycline was i.t. injected to rats after PTX treatment to examine the etiological role of microglial p-ERK expression in PTX induced CINP. (A, B, and D) The protein levels of Iba-1, p-ERK, and EP2 expression in the dorsal spinal cord were determined by western blot analysis. As shown, either PD98059 or minocycline significantly inhibited the expression upregulation of Iba-1, p-ERK, and EP2 after PTX treatment. Data were represented as mean ± SEM. * and ^#^ P＜0.05 for comparison to control and CINP rats, respectively. n = 6 rats for each group. (C) ELISA verified that PD98059 or minocycline also inhibited the increased release of PGE_2_. Data were represented as mean ± SEM. * and ^#^ P＜0.05 for comparison to control and CINP rats, respectively. n = 6 rats for each group. (E) In each time point after PTX treatment, the mechanical withdrawl threshold was compared with one-way ANOVA followed by the Bonferroni post hoc test. * and ^#^ P＜0.05 for comparison to control and CINP rats, respectively. n = 6 rats in each group.

Our previous results showed microglial p-ERK expression and the downstream PGE_2_-EP2 signaling might participate in PTX induced mechanical hypersensitivity, the p-ERK antagonist PD98059 or microglia activation inhibitor minocycline was i.t. injected to rats after PTX treatment to examine the etiological role of microglial p-ERK expression. As shown in Fig. 4**A and B**, PD98059 or minocycline significantly inhibited the expression upregulation of Iba-1 and p-ERK after PTX treatment. Concomitantly, PD98059 or minocycline also inhibited the increased PGE_2_ and EP2 expression, as detected by ELISA and western blot, respectively (Fig. 4**C-D)**.

Additionally, we performed pain behavioral testing in rats after PTX treatment in the presence of i.t. PD98059 or minocycline. As shown in Fig. 4**F**, either PD98059 or minocycline could gradually improve the mechanical hypersensitivity after PTX treatment, indicating an pain relief effect. All of these results showed inhibition of microgalial p-ERK expression could inhibit microglia activation, PGE_2_ and EP2 expression upregulation, as well as ameliorate the mechanical hypersensitivity in CINP rats.

### 3.5 Allopregnanolone level in the dorsal spinal cord is decreased in CINP rats and intragastric administration of exogenous allopregnanolone dose-dependently alleviates PTX-induced mechanical hypersensitivity

**Fig. 5:**
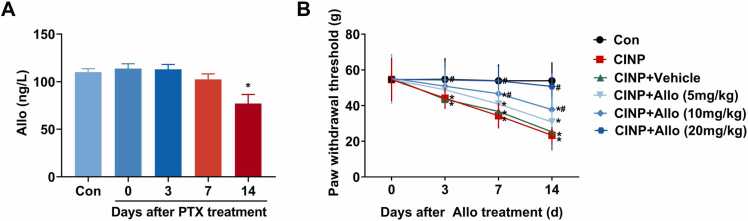
Allopregnanolone level in the spinal cord is decreased in CINP rats and intragastric administration of exogenous allopregnanolone dose-dependently alleviates PTX-induced mechanical hypersensitivity. We established CINP rat model with 4 alternate i.p. injections of PTX 2 mg/kg. Control rats received an equal volume of DMSO. ELISA examined the level of PGE_2_ in the dorsal spinal cord at 0, 3, 7, and 14 day after PTX treatment. (A) We observed a prominent decrease of spinal cord allopregnanolone level at day 14 after PTX treatment. Data were represented as mean ± SEM. *P＜0.05 for comparison to control rats. n = 6 rats for each group. (B) Exogenous allopregnanolone was administered to rats via orogastric tube at doses of 5, 10, or 20 mg/kg once a day on days 1, 3, 5, 7, 9, 11, and 13 after PTX treatment. Pain behavioral testing showed that allopregnanolone dose-dependently improved the mechanical withdrawal threshold in CINP rats. In each time point after PTX treatment, the mechanical withdrawl threshold was compared with one-way ANOVA followed by the Bonferroni post hoc test. * and ^#^ P＜0.05 for comparison to control and CINP rats, respectively. n = 6 rats in each group.

As an neurosteroid having analgesic property, the expression of allopregnanolone in the dorsal spinal cord was detected using ELISA after PTX treatment. As shown in Fig. 5**A**, a prominent decrease of spinal cord allopregnanolone level was observed at day 14 after PTX treatment. Importantly, we tried to examine whether exogenous allopregnanolone supplement could offfer an analgesic effect. As shown in Fig. 5**B**, allopregnanolone dose-dependently improved the mechanical withdrawal threshold in CINP rats, suggesting an analgesic effect of allopregnanolone on PTX-induced mechanical hypersensitivity.

### 3.6 Allopregnanolone dose-dependently alleviates PTX-induced microglial activation, p-ERK, PGE_2,_ and EP2 upregulation, as well as cytokines expression in the dorsal spinal cord in CINP rats

**Fig. 6:**
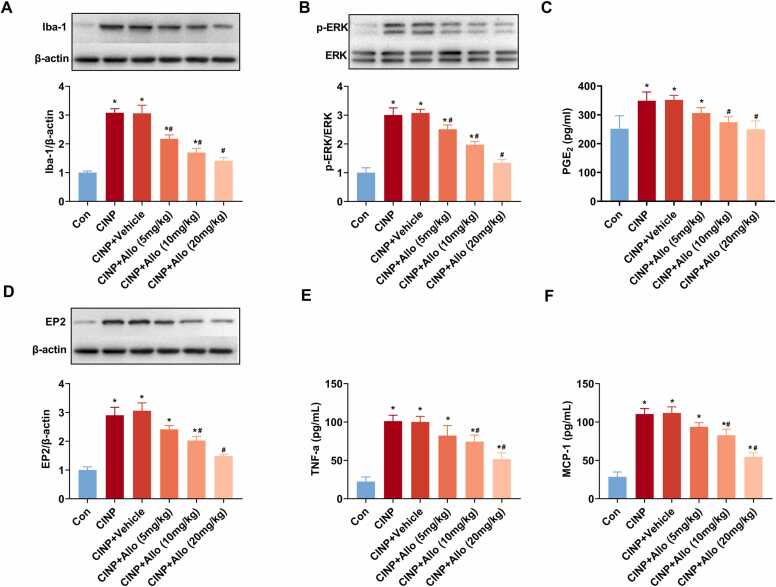
Allopregnanolone dose-dependently alleviates PTX-induced microglial activation, p-ERK, PGE_2_, and EP2 upregulation, as well as cytokines expression in the spinal cord in CINP rats. Exogenous allopregnanolone was administered to rats via orogastric tube at doses of 5, 10, or 20 mg/kg once a day on days 1, 3, 5, 7, 9, 11, and 13 after PTX treatment. (A, B, and D) The protein levels of Iba-1, p-ERK, and EP2 expression in the dorsal spinal cord were determined by western blot analysis. As shown, western blot showed a dose-dependent decrease in Iba-1, p-ERK, and EP2 expression in CINP rats treated with allopregnanolone compared to vehicle-treated CINP rats. Data were represented as mean ± SEM. * and ^#^ P＜0.05 for comparison to control and CINP rats, respectively. n = 6 rats for each group. (C, E, and F) Allopregnanolone dose-dependently reduced PTX-induced PGE_2_, TNF-a, and MCP-1 expression upregulation in the dorsal spinal cord in CINP rats as detected by ELISA. * and ^#^ P＜0.05 for comparison to control and CINP rats, respectively. n = 6 rats for each group.

Our previous results showed exogenous allopregnanolone could ameliorate PTX-induced mechanical hypersensitivity, we further explored whether PTX-induced microglial activation, p-ERK upregulation, as well as PGE_2_-EP2 signaling participated in the observed analgesic effect. As shown in Fig. 6**A-D**, western blot or ELISA showed a dose-dependent decrease in Iba-1, p-ERK, PGE2 and EP2 expression in CINP rats treated with allopregnanolone compared to vehicle-treated CINP rats. Further, allopregnanolone dose-dependently reduced PTX-induced TNF-a and MCP-1 expression upregulation in the dorsal spinal cord in CINP rats (Fig. 6**E-F)**. Accordingly, we proposed that the microglial p-ERK expression and PGE_2_-EP2 mediated migroglia-neurons signaling participated in the analgesic effect of allopregnanolone for PTX-induced mechanical hypersensitivity.

### 3.7 Subcutaneous allopregnanolone synthesis inhibitor medroxyprogesterone further reduces endogenous allopregnanolone and blocks all effects of allopregnanolone in CINP rats

**Fig. 7:**
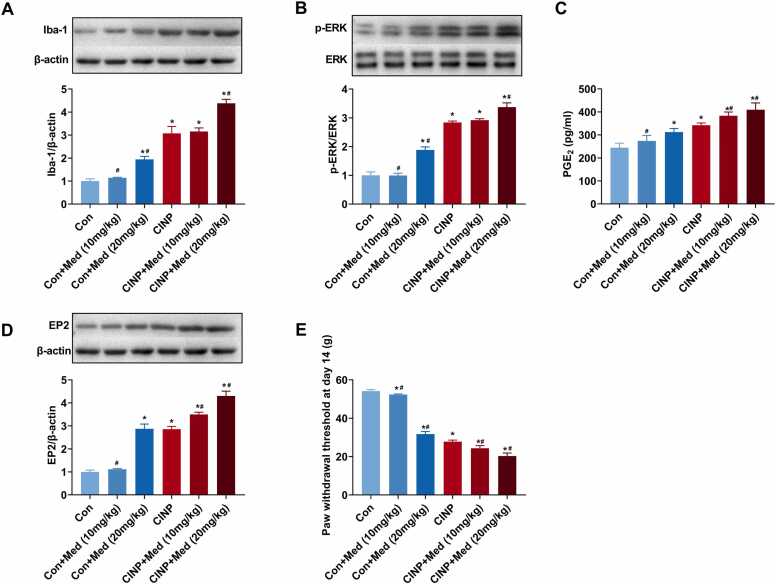
Subcutaneous allopregnanolone synthesis inhibitor medroxyprogesterone (Med) further reduces endogenous allopregnanolone and blocks all effects of allopregnanolone in CINP rats. The allopregnanolone synthesis inhibitor medroxyprogesteron was injected subcutaneously into CINP rats at doses of 10 or 20 mg/kg on days 1, 3, 5, 7, 9, 11, and 13 after PTX treatment to further reduce the endogenous allopregnanolone. (A, B, and D) The protein levels of Iba-1, p-ERK, and EP2 expression in the dorsal spinal cord were determined by western blot analysis. As shown, we observed a significant increase in Iba-1 and p-ERK expression in the dorsal spinal cord of CINP rats receiving medroxyprogesteron. Data were represented as mean ± SEM. * and ^#^ P＜0.05 for comparison to control and CINP rats, respectively. n = 6 rats for each group. (C) As detected by ELISA, we observed a significant increase in the PGE_2_ expression in the dorsal spinal cord in CINP rats treated with 10 or 20 mg/kg medroxyprogesterone. * and ^#^ P＜0.05 for comparison to control and CINP rats, respectively. n = 6 rats for each group. (E) Pain behavioral testing showed at day 14 after PTX treatment, mechanical withdrawal threshold was further decreased if treated with medroxyprogesterone at doses of 10 or 20 mg/kg. * and ^#^ P＜0.05 for comparison to control and CINP rats, respectively. n = 6 rats in each group.

Our previous results suggested microglial p-ERK expression and PGE_2_-EP2 mediated migroglia-neurons signaling participated in the analgesic effect of allopregnanolone for PTX-induced mechanical hypersensitivity. To validate these results, the allopregnanolone synthesis inhibitor medroxyprogesteron was injected subcutaneously into CINP rats to further reduce the endogenous allopregnanolone. Microglial activation, p-ERK, PGE_2_, and EP2 expression, as well as pain behavior were examined. As shown in Fig. 7**A-B**, western blot analysis showed significant increase in Iba-1 and p-ERK expression in the dorsal spinal cord of CINP rats receiving medroxyprogesteron 20 mg/kg, but not 10 mg/kg.

Additionally, we observed a significant increase in the PGE_2_ and EP2 expression in the dorsal spinal cord in CINP rats treated with 10 or 20 mg/kg medroxyprogesterone **(**Fig. 7**C-D)**. At day 14 after PTX treatment, mechanical withdrawal threshold was further decreased if treated with medroxyprogesterone at doses of 10 or 20 mg/kg **(**Fig. 7**E)**.

## 4 Discussion

This study indicates that the natural neurosteroid allopregnanolone presents an analgesic effect for PTX induced mechanical hypersensitivity in rats, partially via inhibiting the spinal dorsal horn PGE_2_-EP2 mediated microglia-neuron signaling. Exogenous allopregnanolone dose-dependently ameliorated PTX-induced microglial activation, p-ERK, PGE_2,_ and EP2 upregulation, reduced PTX-induced cytokines expression in the dorsal spinal cord, and relieved PTX-induced mechanical hypersensitivity in CINP rats. In addition, after subcutaneous injection of the allopregnanolone synthesis inhibitor medroxyprogesteron, the increased p-ERK, PGE_2_ and EP2 expression in CINP rats were further upregulated, and the mechanical withdrawal threshold were further decreased in rats.

After peripheral nerve injury, ERK phosphorylation in the spinal cord is a common phenomenon. In Dr. Zhuang’s study (Zhuang et al., 2005), p-ERK expression upregulation was sequentially observed in the ipsilateral superficial dorsal horn neurons, microglia, microglia and astrocytes, or astrocytes within 6 hours, at days 1–3, day 10, and day 21 after L5 spinal nerve ligation, respectively. In our study, ERK phospholyation was observed in the microglia at day 14 after PTX treatment, but not neurons or astrocytes. Additionally, PGE_2_ is a crucial signaling molecule involved in allodynia and hyperalgesia (Vieira et al., 2016, St-Jacques and Ma, 2014). The MAPK/ERK pathway was required for the generation of PGE_2_ in human chondrocytes (Zhao et al., 2007, Wang et al., 2010). At day 14 after PTX treatment, we tentatively examined PGE_2_ expression in the dorsal spinal cord. As expected, the level of PGE_2_ in the dorsal spinal cord increased significantly after PTX treatment.

The action of PGE_2_ are generated by G-protein-coupled EP receptors, including EP1, 2, 3, and 4 (Bär et al., 2004). In vitro studies proved microglia synthesize and release PGE_2_ (Zhang et al., 2006, Ikeda-Matsuo et al., 2005, Nakano et al., 2008). Meanwhile, PGE_2_ increases the excitability of dorsal horn neurons and directly activates neurons through EP2 receptors (Baba et al., 2001). Deficiency in EP2 receptor completely blocks spinal PGE_2_ evoked hyperalgesia. In our study, an upregulation of EP2 expression was observed at day 14 after PTX treatment. Such EP2 expression upregulation occurred in neurons but not microglia or astrocytes. To further elucidate the contribution of the spinal cord PGE_2_-EP2 mediated microglia-neuron signaling in PTX induced mechanical hypersensitivity, p-ERK inhibitor PD98059 or microglia inhibitor minocycline was intrathecal injected in CINP rats. As shown by Fig. 4, either PD98059 or minocycline could inhibit Iba-1 expression, decrease p-ERK, PGE2, and EP2 upregulation, and finally improve PTX induced mechanical hypersensitivity.

Allopregnanolone regulates various receptors or channels that are distributed in the pain regulatory pathway, such as GABA_A_ and glycine receptors, as well as L-and T-calcium channels, in an autocrine or paracrine manner (Belelli and Lambert, 2005, Mitchell et al., 2007, Pathirathna et al., 2005). Consequently, diverse mechanisms underlying allopregnanolone’s analgesic effect have been proposed. Allopregnanolone reversed the down-regulation of GABA_A_ receptors in the spinal cord in the diabetic neuropathic pain rat model (Afrazi and Esmaeili-Mahani, 2014). Besides, allopregnanolone could alleviate the activation of glial ERK through GABA_A_ receptors in the cuneate nucleus to alleviate median nerve injury induced mechanical hypersensitivity (Huang et al., 2016). Further, in cultured Xenopus oocytes, allopregnanolone inhibited voltage-gated sodium channels that contain Na_V_1.3α subunit in a concentration-dependent manmer (Horishita et al., 2018). Furthermore, via reducing caspase 3 activation and the Bax/Bcl2 ratio, allopregnanolone alleviated high glucose-induced neuropathic pain via preventing high glucose-induced apoptosis in PC12 cells (Afrazi et al., 2014). Of note, allopregnanolone was reported to prevent and suppress oxaliplatin induced painful neuropathy via improving sciatic nerve conduction velocity and action potential peak amplitude, as well as restoring intra-epidermal nerve fiber control density in DRG neurons and sciatic nerve axons (Meyer et al., 2011). However, the mechanism of allopregnanolone in reliving CINP remains unclear.

Dr. Kawano found that the spinal cord and brain of rats with spinal nerve ligation had a higher level of allopregnanolone than those of control rats (Kawano et al., 2011). Consistently, in Dr. Patte-Mensah’s time-course study, after sciatic nerve ligation, endogenous allopregnanolone significantly increased at day 2, peaked at day 6, and then gradually decreased at day 10 after nerve injury (Patte-Mensah et al., 2004). However, in our study, the expression of allopregnanolone decreased in the spinal cord at days 7–14 after PTX treatment. We considered there might be two reasons contributing to such a discrepancy. Firstly, the animal model in our study and others’ were different. Secondly, we used ELISA to examine allopregnanolone concentration, while radioimmunoassay, as well as radioimmunoassay and high performance liquid chromatography, were used in Dr. Kawano’s and Dr. Patte-Mensah’s study, respectively.

To explore the analgesic effect of allopregnanolone for CINP, various doses of exogenous allopregnanolone were administered in CINP rats at days 1, 3, 5, 7, 9, 11, and 13 after PTX treatment. As shown in Fig. 5, allopregnanolone dose-dependently upregulated PTX induced decrease in mechanical withdrawal threshold, showing an analgesic effect of allopregnanolone. Mechanistically, allopregnanolone dose-dependently down-regulated Iba-1 expression, decreased the levels of p-ERK, PGE2, and EP2, and decreased the expression of the cytokines TNF-α and MCP-1.

TNF-α and MCP-1 are two essential cytokines that regulate neuronal excitability and are involved in the induction and maintenance of neuropathic pain (Liu et al., 2019, Yan et al., 2019). Electrophysiologic studies have shown TNF-α enhances the frequency of spontaneous EPSCs and enhances AMPA- or NMDA-induced current, thus promoting excitatory synaptic transmission in superficial dorsal horn neurons (Kawasaki et al., 2008). Intrathecal injection of TNF-α or spinal nerve ligation produces JNK-dependent pain hypersensitivity. TNF-α also activates c-Jun N-terminal kinase via TNF receptor-1 in cultured astrocytes, and the TNF-α/JNK pathway strongly induces MCP-1 expression. Spinal injection of the MCP-1 neutralizing antibody could attenuate spinal nerve ligation induced neuropathic pain (Gao et al., 2009). In our study, following PTX treatment, there was an upregulation of both TNF-α and MCP-1 in the dorsal spinal cord of CINP rats. And exogenous allopregnanolone dose-dependently reduced the levels of TNF-α and MCP-1 in the dorsal spinal cord of CINP rats, showing an analgesic effect.

Additionally, we reduced the allopregnanolone level in the spinal cord with medroxyprogesteron. As an inhibitor of 3α-hydroxysteroid oxidoreductase (3α-HSOR), medroxyprogesteron reduces the expression of allopregnanolone by preventing the conversion of dihydroprogesterone to allopregnanolone (Qiu et al., 2015). As shown, after subcutaneous injection of 10 or 20 mg/kg medroxyprogesteron, we observed that 20 mg/kg medroxyprogesteron prominently increased Iba-1 expression, increased the levels of p-ERK, PGE_2_, and EP2, and finally worsened the mechanical pain. All of these results indicated that the analgesic effect of allopregnanolone on PTX induced mechanical hypersensitivity might partially involve the spinal cord PGE_2_-EP2 mediated microglia-neuron signaling.

However, we need to acknowledge that there are several limitations of this study. Firstly, we only observed the cellular distribution of p-ERK on day 14 rather a dynamic distribution after PTX treatment. Secondly, other than EP2 receptor, it was reported that inflammatory pain and nociceptor sensitization are mediated by PGE_2_/EP4 signaling-induced EP4 externalization in DRG neurons (St-Jacques and Ma, 2013). The role of other EP receptors is still unclear. Thirdly, other than inhibiting allopregnanolone production, medroxyprogesteron is also a positive modulator of specific GABA_A_ receptor subtypes (Roshni et al., 2022, Karen et al., 2009), rendering the explanation of the pro-nociceptive effect of medroxyprogesterone in our study more complex. Therefore, more studies are warranted to clarify these points.

## 5 Conclusion

Our results indicate that the natural neurosteroid allopregnanolone presents an analgesic effect for PTX-induced mechanical hypersensitivity, partially via inhibiting the dorsal spinal cord PGE_2_-EP2 mediated microglia-neuron signaling, providing an alternative explanation for the analgesic effect of allopregnanolone for CINP.

## CRediT authorship contribution statement

**Kunlin Guo:** Writing – original draft, Formal analysis, Data curation. **Lei Gao:** Software, Methodology, Investigation. **Ping Li:** Software, Investigation. **Liping Zhao:** Validation, Software, Project administration. **Shanwu Feng:** Supervision, Resources, Data curation. **Xian Wang:** Writing – review & editing, Funding acquisition, Conceptualization.

## Declaration of Competing Interest

The authors declare that they have no known competing financial interests or personal relationships that could have appeared to influence the work reported in this paper.

## Data Availability

The data that support the findings of this study are available from the corresponding author upon reasonable request.
